# Serendipitous Stimulation of Nucleus Basalis of Meynert—The Effect of Unintentional, Long-Term High-Frequency Stimulation on Cognition in Parkinson’s Disease

**DOI:** 10.3390/jcm11020337

**Published:** 2022-01-11

**Authors:** I. Daria Bogdan, D. L. Marinus Oterdoom, Teus van Laar, Rients B. Huitema, Vincent J. Odekerken, Judith A. Boel, Rob M. A. de Bie, J. Marc C. van Dijk, on behalf of the NSTAPS Study Group

**Affiliations:** 1 Department of Neurosurgery, University Medical Center Groningen, University of Groningen, 9713 Groningen, The Netherlands; i.bogdan@maastrichtuniversity.nl (I.D.B.); j.m.c.van.dijk@umcg.nl (J.M.C.v.D.); 2 Department of Neurology, University Medical Center Groningen, University of Groningen, 9713 Groningen, The Netherlands; t.van.laar@umcg.nl (T.v.L.); r.b.huitema@umcg.nl (R.B.H.); 3 Department of Neurology, Amsterdam Neuroscience Institute, Amsterdam University Medical Center, 1105 Amsterdam, The Netherlands; v.j.odekerken@amsterdamumc.nl (V.J.O.); judithboel@hotmail.com (J.A.B.); r.m.debie@amsterdamumc.nl (R.M.A.d.B.)

**Keywords:** Parkinson’s disease, Parkinson’s disease dementia, cognitive impairments, cognitive function, deep brain stimulation

## Abstract

There is a growing interest in deep brain stimulation (DBS) of the nucleus basalis of Meynert (NBM) as a potential therapeutic modality for Parkinson’s disease dementia (PDD). Low-frequency stimulation has yielded encouraging results in individual patients; however, these are not yet sustained in larger studies. With the aim to expand the understanding of NBM-DBS, we share our experience with serendipitous NBM-DBS in patients treated with DBS of the internal Globus pallidus (GPi) for Parkinson’s disease. Since NBM is anatomically located ventral to GPi, several GPi-treated patients appeared to have the distal contact of DBS-electrode(s) positioned in the NBM. We hypothesized that unintentional high-frequency NBM-DBS over a period of one year would result in the opposite effect of low-frequency NBM-stimulation and cause cognitive decline. We studied a cohort of 33 patients with bilateral high-frequency DBS in the GPi for Parkinson’s disease, of which twelve were unintentionally co-stimulated in NBM. The subgroups of unintentional unilateral (*N* = 7) and bilateral NBM-DBS (*N* = 5) were compared to the control group of bilateral GPi-DBS (*N* = 11). Here, we show that unintentional high-frequency NBM-DBS did not cause a significantly faster decline in cognitive function. Further research is warranted for characterizing the therapeutic role of NBM-DBS.

## 1. Introduction

Parkinson’s disease (PD) is the fastest growing neurological disorder in the world [1]. Parkinson’s disease dementia (PDD) is diagnosed in the vast majority of PD patients during the disease course [2,3]. Clinically, PDD can be characterized as a dysexecutive syndrome with impairments in attention, executive and visuospatial functions, as well as moderately impaired memory and behavioral symptoms such as apathy and psychosis [4]. Pharmacotherapeutic options are limited to cholinesterase inhibitors and memantine and offer only modest and often non-sustained effects. Deep brain stimulation (DBS) as treatment for cognitive decline in PDD is a subject of ongoing interest [5]. A promising target is the nucleus basalis of Meynert (NBM) due to its widespread cholinergic innervation of the cortex (for a review of the NBM functional anatomy and evidence for involvement in the cognitive decline in PDD, see Gratwicke et al., 2013) [6]. NBM holds a pivotal role in a range of cognitive functions, including those commonly affected in PDD (arousal, attention, perception, and memory) [7]. This is in line with the tight correlation observed between the extent of NBM degeneration and cortical cholinergic deficits and cognitive decline [8]. According to pilot investigations, NBM-DBS may be considered a safe procedure, without significant stimulation-induced side effects. Evidence regarding its clinical significance, however, has been equivocal (Table 1). 

Namely, while individual patients treated with low-frequency NBM-stimulation have shown encouraging results [9,10,14], larger trials yielded modest results at most [11,13]. The varied results might be attributed to several factors, including suboptimal NBM targeting, given its irregular anatomical shape [10,11] and its cytochemical heterogeneity [15]. The use of predefined stimulation parameters might have also played a detrimental role. Although the interaction of stimulation parameters with the stimulation substrate has yet to be elucidated, evidence suggests that DBS-optimization might require broad parameter searches, extending beyond the limits of conventional stimulation parameters (i.e., preset pulse-widths and frequencies) [16]. In line with this, Bergfeld and colleagues underline the importance of first ensuring optimal DBS titration before establishing its effectivity in a randomized clinical trial of DBS for treatment-resistant depression [17]. Patient selection has also been proposed as a putative prediction factor, with recent observations suggesting that DBS may be more effective in patients with milder impairment, e.g., mild cognitive impairment or mild AD, compared to those with more advanced stages of AD [18]. Addressing these factors, although a challenging feat, will be crucial in the endeavor to establish the role of NBM-DBS in memory and cognitive deficits.

With the scope of expanding the current understanding of NBM-DBS, as well as guiding future research, we share our experience with serendipitous NBM-DBS in patients treated with GPi-DBS for PD. Since NBM is anatomically located ventrally to GPi, several GPi-treated patients turned out to have the distal contact of the DBS-electrode(s) positioned in the NBM. Here, we present the effect of unintentional, long-term high-frequency stimulation on cognition in PD. Moreover, we challenge the hypothesis that continuous, high-frequency (NBM-)stimulation would create an *informational lesion* [19,20] and, thus, worsen cognition [21]. 

## 2. Materials and Methods

### 2.1. Study Design and Participants 

**Figure 1:**
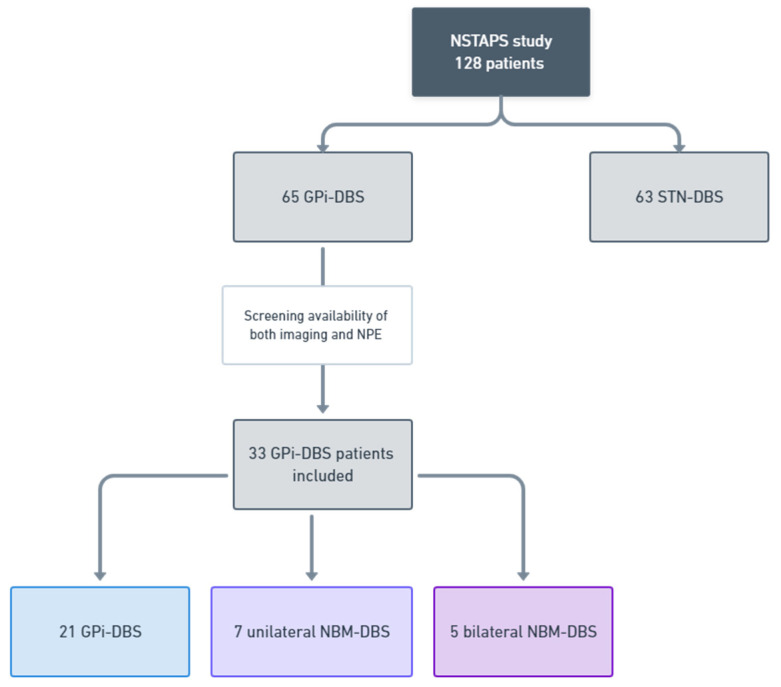
Data collection. Between January 2007 and March 2011, 128 patients participated in the NSTAPS study. Sixty-five patients were randomized to receive GPi-DBS. Since NBM is anatomically located ventral to the GPi, several GPi-treated patients appeared to have the distal contact of the DBS-electrode(s) positioned in NBM. The research database was screened for the concurrent presence of neuroimaging and neuropsychological evaluations (NPE), which were available for thirty-three GPi-DBS candidates. The positions of the DBS electrodes and active contacts were reviewed in these patients, which yielded three categories: GPi-DBS (*N* = 11), unilateral NBM-DBS (*N* = 7), and bilateral NBM-DBS (*N* = 5). Abbreviations: *NPE* = neuropsychological evaluations.

Between January 2007 and March 2011, 128 patients participated in The Netherlands SubThalamic and Pallidal Stimulation (NSTAPS) study. Enrollment criteria, study design, and methods are described elsewhere [22]. Following randomization, 65 patients underwent GPi-DBS (Figure 1). Seven patients did not complete the neuropsychological assessment at the 12-month follow-up. Of the remaining 58 patients, neuroimaging was available in 25 patients. To ascertain the position of the DBS electrodes, the preoperative 3T-MRI (Philips Intera, Eindhoven, The Netherlands) and post-operative CT (Sensation 64, Siemens, Erlangen, Germany) scans were merged with BrainLAB-software (BrainLAB, Heimstetten, Germany). The NBM was demarcated according to the Atlas for Stereotaxy of the Human Brain [23]. Projections of the DBS-electrode contacts were characterized as follows: (1) both electrodes solely in the GPi, no contact with NBM; (2) unilateral active contact point located inside the NBM (unilateral NBM-DBS); (3) bilateral active contact points located in the NBM (bilateral NBM-DBS). Cognitive outcomes from the neuropsychological assessment were compared between the three subgroups. 

### 2.2. Neuropsychological Examination 

All patients underwent neuropsychological examinations (NPE) during the on-drug phase at baseline and at one year after implantation, with the DBS-system switched on. NPE covered the following cognitive domains: memory, speed of information processing, attention and working memory, language, and executive functions. Verbal memory, both immediate and delayed recall, was assessed with the Dutch version of Rey’s Auditory Verbal Learning Test (AVLT) and the Rivermead Behavioural Memory Test (RBMT). For the assessment of speed of information processing, attention and working memory, the Single Choice Reaction Time Measurement of Vienna Test System (VTS-RT1), the Stroop Color-Word test (Stroop), the Trail-Making Test part A (TMT-A), and the subtest Digit Span of the Wechsler Adult Intelligence Scale III (DS) were used. The naming of words in a semantic category, as part of the Controlled Oral Word Association Test (COWAT), was used to assess semantic fluency in the language domain (COWAT-SF). Trail-Making Test part B (TMT-B) was used to assess cognitive flexibility. The naming of words starting with a specific letter, also part of the COWAT, was used to assess phonetic fluency (COWAT-PF). Raw test scores were normalized for age, gender, or education if needed and transformed to T-scores.

### 2.3. Statistical Analysis 

Data were tested for normality by using the Kolmogorov–Smirnov test. The difference in cognitive performance between baseline and at 1 year after implantation was assessed between three subgroups by means of repeated-measures ANOVA (main effect group, main effect pre-post, and interaction effect group × pre-post). In order to correct for any discrepancies in the length of the follow-up interval, the number of days between two assessments was entered as covariate. Statistical analysis was performed using SPSS (SPSS IBM version 28.0, New York, NY, USA).

## 3. Results

### 3.1. Patient Characteristics and DBS Targets 

**Figure 2:**
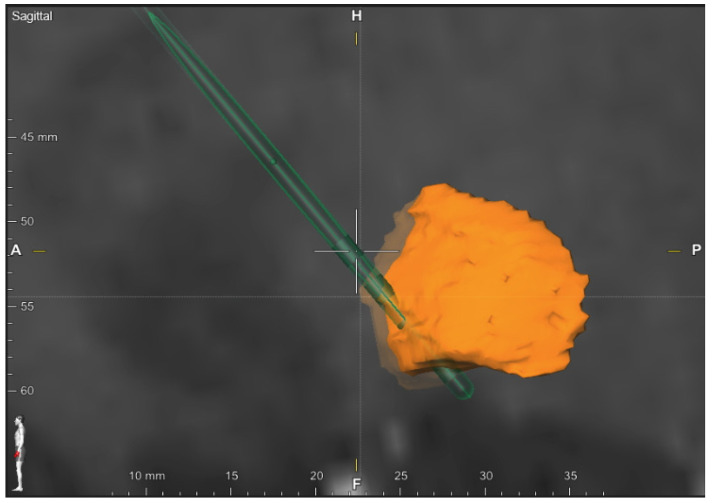
Review of the DBS-target. Sagittal view of a Gpi electrode crossing the Gpi. Patient was stimulated on the most distal contact point. Coordinates relative to anterior commissure: 18.3 mm lateral, 6.5 mm posterior, and 6.0 mm inferior. Stimulation settings: 2.4 Volt, frequency 130Hertz, pulse width 60 microseconds (A: anterior, P: posterior).

Both neuroimaging and neuropsychological data were available for 33 patients (58.4 ± 7.8 years; six women). Fused MRI and CT scans were reviewed, as well as the active electrode contacts, to ascertain the DBS-target (Figure 2). Twenty-one patients were classified as receiving GPi-DBS, seven patients received unilateral NBM-DBS, and the remaining five patients were stimulated bilaterally in NBM. Patient characteristics are presented in Table 2. The three groups did not differ on any variables at baseline: age (F(2,30) = 1.371, *p* = 0.26); gender (χ(2) = 0.093, *p* = 0.95); disease duration (H(2) = 2.434, *p* = 0.29); age at diagnosis (F(2,30) = 1.06, *p* = 0.35); age at DBS-surgery (F(2,30) = 1.52, *p* = 0.23); number of days elapsed from baseline to follow-up (F(2,29) = 2.464, *p* = 0.103); voltage (F(2,29) = 1.29, *p* = 0.28); frequency (χ(2) = 0.06, *p* = 0.96); and pulse width (χ(2) = 1.04, *p* = 0.59). 

### 3.2. Neuropsychological Outcomes 

Repeated-measures ANOVA showed a significant main pre-post effect for Stroop word (F(1,28) = 5.807; *p* = 0.23), TMTA (F(1,28) = 6.031; *p* = 0.02), and TMTB/TMTA (F(1,28) = 10.008; *p* = 0.004), but no significant main effects were observed for the group on any of the variables. Most importantly, no significant interaction effect (group × pre-post) on any of the variables was found. In Table 3, mean values and *p*-values of the interaction effect are reported.

## 4. Discussion

In this study, we explored the post-hoc hypothesis that serendipitous high-frequency stimulation of the NBM might have a negative impact on cognitive functioning in affected subgroups. Although a general decline in some of the cognitive domains was found, no difference in decline between the GPi-stimulated and NBM-stimulated groups was observed. According to these findings, long-term high-frequency NBM-stimulation does not appear to have a negative impact on cognition in PD-patients. 

A possible explanation of the lack interference with cognitive functioning could be related to the direction of targeting NBM via the GPi, which provides an almost vertical approach to the flat, disc-like structure of the NBM. This might have less influence on the NBM output than stimulation in a horizontal plane. On the other hand, diffusion-weighted imaging-based tractography (DTI) has helped refine DBS targeting and modulating white-matter tracts is increasingly favored over brain nuclei [24,25]. So far, two studies have used DTI to track NBM cholinergic pathways [26,27]. Both models successfully revealed tracts in both medial and lateral pathways, which is line with previous (immuno-)histochemical studies [28]. Correspondingly, a functional resting-state magnetic resonance imaging (rs-fMRI) study in healthy adult individuals revealed two distinct anterior-medial and posterior-lateral clusters [29]. Notably, the two clusters show largely different functional connectivity profiles, namely, the (1) anterior-medial cluster is connected to the hippocampus and interconnected nodes of an extended medial cortical memory network, and the (2) posterior-lateral cluster is connected to the anterior insula and dorsal anterior cingulate components of a salience/attention network. New insights obtained by combining electrode location reconstructions and tractography studies are refining the concept of the neuromodulation substrate from the former disease-specific networks to the more focused symptom-specific networks [30]. As such, NBM-DBS might specifically require targeting the corresponding white matter tracts required to modulate memory and/or attention. Targeting NBM tracts rather than its grey matter might also be supported by the observation that (1) the coherence with the temporal region was of a smaller magnitude in the NBM region compared to outside of it and that (2) despite established connections of the NBM with many cortical regions, coherence only with the temporal region was observed inside the nucleus [31]. These pilot results might have reflected cholinergic deterioration congruent with PDD and should, thus, be interpreted accordingly. Namely, even though these findings might not support the lack of cognitive interference in our patients (who had a relatively conserved NBM-cytoarchitecture), this remains a possibly crucial consideration for surgical targeting in PDD patients. Apart from spatial targeting, the temporal specificity of the delivered neuromodulation must also be considered. For instance, delivering stimulation in phase with a rhythm may amplify it, while delivering it not-in-phase may either cancel or attenuate it [32]. To add another layer of complexity to temporal targeting, evidence suggests that different aspects of cognition may be encoded in different oscillatory frequencies [33]. Open-loop NBM-DBS may, thus, fail to interact purposefully with networks underlying memory and cognition. Novel approaches employing closed-loop neuromodulation for treatment-resistant depression [34] and enhancement of cognitive control [35] are slowly emerging and may offer valuable insights for individualizing NBM-DBS. A pressing challenge that may aid problems is identifying a biomarker for cognitive functioning, which could allow refining stimulus delivery. The latter is additionally important in light of the responsibility towards patients with implants, where “a failure to explore the many combinatorial possibilities that could still be tried, once an implanted device is already in place, seems to us a breach of the ethical doctrine of proportionality” [36,37].

## 5. Limitations 

The fact that the NBM was not intentionally targeted might be considered a limitation of this study. Nevertheless, the position of the active contact point of the DBS-electrode in relation to the NBM was carefully assessed. Given the hitherto lack of a reliable volume of tissue activated (VTA) approximation algorithm [38], the position of the active contact was ascertained visually against the anatomical background. Although this allowed the identification of patients receiving NBM-DBS, it might not have definitely excluded patients receiving GPi-DBS, with current spread extending to the NBM. However, the observation that simultaneous GPi-NBM stimulation showed improved neuropsychological measurement in one patient with similar surgical targeting may discourage that possibility [12]. Another limitation is that we were not able to explore the effects of low-frequency stimulation in our patients. Moreover, from the limited available data, it is not possible to exclude with certainty a masked effect of NBM-DBS due to medication. Lastly, the current study is an explorative, post-hoc analysis of a subgroup of the NSTAPS-trial. As such, the study lacks a priori power analysis to confidently exclude a significant detrimental effect of high-frequency NBM-DBS. Nevertheless, by scrutinizing electrode positions of patients who underwent DBS surgery, we were able to add a considerable number of NBM-stimulated patients to the literature and, thus, expanded the knowledge on its effects.

## 6. Conclusions

In this post-hoc analysis of a subgroup of the NSTAPS-trial, we conclude that after one-year follow-up, unintentional high-frequency NBM-stimulation does not result in a statistically significant decline in cognitive function of PD-patients. Individualizing patient selection, as well as the spatiotemporal coordinates of NBM-DBS, will be essential in establishing the therapeutic role of NBM-DBS in the treatment of PDD. 

## Figures and Tables

**Table 1:** Outcomes of NBM-DBS.

Group	Study Design	*N*	Diagnosis	DBS Target(s)	NBM-Targeting	Stimulation	Outcomes
Freund et al., 2009 [9]	Individual clinical trial	1	PDD	Bilateral STN-DBS and NBM-DBS	Ch4 intermedius via deep frontolateral approach	LFS Sham	“Clear improvements in various aspects of cognitive functioning.”
Kuhn et al., 2015 [10]	RCT followed by open-label	6	AD	Bilateral NBM-DBS	Ch4 division of the NBM	LFS Sham	“On the basis of stable/improved primary outcome parameters 12 months after surgery, 4/6 patients were considered responders.”
Gratwicke et al., 2018 [11]	RCT, doubleblind crossover	6	PDD	Bilateral NBM-DBS	Ch4i subsector via more posterior entry point than used for conventional STN-DBS	LFS Sham	“ […] the range of cognitive deficits were not consistently improved.”
Nombela et al., 2019 [12]	Individual clinical trial	1	PD-MCI	Bilateral GPi-NBM-DBS	NBM complex but not in the Ch4 intermedius	LFS	“[…] improvements were noted in all the neuropsychological measurements except for the Categorical Verbal Fluency and Reverse Digit Span subscale”
Gratwicke et al., 2020 [13]	RCT, doubleblind crossover	6	DLB	Bilateral NBM-DBS	Ch4i subsector via a frontal entry point, on/posterior to the coronal suture	LFS Sham	“No consistent improvements were observed in exploratory clinical outcome measures.”
Zhang et al., 2021 [14]	Individual clinical trial	1	AD	Bilateral NBM-DBS	Ch4p area	LFS	“improvement in ADAS-cog, […], executive functions”, however, according to his caregiver ”no substantial changes during daily life”

Abbreviations: AD = Alzheimer’s disease; DLB = Dementia with Lewy bodies; GPi = internal globus pallidus; LFS = low-frequency stimulation; MCI = mild cognitive impairment; NBM = nucleus basalis of Meynert; PDD = Parkinson’s disease dementia; STN = subthalamic nucleus.

**Table 2:** Baseline clinical characteristics of the study sample.

Patient	Age	Gender	Disease Duration	Age at Diagnosis	Age at Surgery	Interval FU (Days)	Electrode Montage (Left/Right)	Stimulation Parameters (Voltage, Frequency, Pulse Width)
GPi-DBS *N* = 21								
PD1	60	Male	16	44	61	373	unipolar/unipolar	2.4 V, 130 Hz, 90 μs
PD2	57	Male	10	52	57	524	bipolar/bipolar	2.0 V, 130 Hz, 60 μs
PD3	63	Male	10	54	64	483	bipolar/unipolar	2.8 V, 130 Hz, 90 μs
PD4	65	Male	13	53	66	427	unipolar/unipolar	1.8 V, 130 Hz, 60 μs
PD5	66	Female	10	58	67	455	bipolar/bipolar	2.8 V, 185 Hz, 60 μs
PD6	71	Male	11	61	72	405	unipolar/unipolar	3.5 V, 130 Hz, 60 μs
PD7	64	Female	19	51	65	413	unipolar/unipolar	3.5 V, 130 Hz, 60 μs
PD8	67	Male	20	48	67	421	unipolar/unipolar	3.0 V, 130 Hz, 60 μs
PD9	60	Male	9	51	60	472	unipolar/unipolar	3.3 V, 130 Hz, 60 μs
PD10	62	Male	8	54	62	398	bipolar/bipolar	3.0 V, 130 Hz, 60 μs
PD11	54	Male	12	43	55	393	unipolar/unipolar	1.5 V, 130 Hz, 60 μs
PD12	50	Male	14	37	51	392	unipolar/unipolar	3.6 V, 130 Hz, 60 μs
PD13	61	Female	17	45	62	370	unipolar/unipolar	2.5 V, 130 Hz, 60 μs
PD14	58	Male	14	44	58	360	unipolar/bipolar	2.0 V, 130 Hz, 90 μs
PD15	68	Male	10	59	68	427	bipolar/bipolar	3.5 V, 135 Hz, 90 μs
PD16	60	Male	7	54	60	455	unipolar/unipolar	3.5 V, 135 Hz, 90 μs
PD17	66	Male	19	50	67	189	unipolar/unipolar	2.5 V, 135 Hz, 60 μs
PD18	54	Male	11	45	55	428	unipolar/unipolar	2.4 V, 135 Hz, 120 μs
PD19	58	Male	15	44	58	439	unipolar/unipolar	3.0 V, 135 Hz, 90 μs
PD20	56	Male	10	47	57	412	bipolar/bipolar	1.5 V, 130 Hz, 60 μs
PD21	43	Female	4	40	43	421	unipolar/unipolar	3.3 V, 135 Hz, 90 μs
Unilateral NBM-DBS *N* = 7								
PD22	69	Male	10	59	69	573	bipolar/bipolar	3.5 V, 185 Hz, 90 μs
PD23	50	Female	8	42	50	457	unipolar/unipolar	2.4 V, 130 Hz, 60 μs
PD24	58	Male	10	48	58	545	bipolar/bipolar	2.0 V, 130 Hz, 60 μs
PD25	65	Male	11	64	65	393	unipolar/unipolar	3.6 V, 130 Hz, 60 μs
PD26	59	Male	5	54	60	401	unipolar/unipolar	3.3 V, 130 Hz, 60 μs
PD27	36	Male	7	30	37	364	unipolar/unipolar	3.5 V, 130 Hz, 60 μs
PD28	51	Male	17	36	52	495	bipolar/unipolar	2.8 V, 135 Hz, 60 μs
Bilateral NBM-DBS *N* = 5								
PD29	64	Female	10	54	64	406	unipolar/unipolar	3.5 V, 130 Hz, 60 μs
PD30	61	Male	8	53	61	608	bipolar/bipolar	3.2 V, 130 Hz, 90 μs
PD31	46	Male	11	35	46	385	bipolar/bipolar	3.3 V, 130 Hz, 60 μs
PD32	57	Male	24	35	58	unknown	bipolar/unipolar	3.5 V, 135 Hz, 90 μs
PD33	50	Male	11	39	50	554	unknown	unknown

Abbreviations: *HFS* = high-frequency stimulation (the stimulation frequency was 130 Hz in all patients); *Interval FU* = interval to follow-up (the number of days elapsed from the baseline measurements until the follow-up measurements).

**Table 3:** Neuropsychological outcomes at baseline and following one year of DBS.

	Baseline (PRE)			One-Year Follow-Up (POST)			*p* Value Group × Pre-Post
	GPi-DBS	Unilateral NBM-DBS	Bilateral NBM-DBS	GPi-DBS	Unilateral NBM-DBS	Bilateral NBM-DBS	
Verbal Memory							
AVLT immediate recall	48.09 ± 10.88	46.85 ± 11.49	51 ± 10.07	43.09 ± 9.85	44.85 ± 13.55	44.4 ± 7.82	0.91
AVLT delayed recall (relative to IR)	45.85 ± 9.06	47.42 ± 11.44	51 ± 7	41.85 ± 11.2	42.42 ± 10.13	52.6 ± 11.67	0.31
RBMT immediate	41.76 ± 13.78	37.14 ± 10.41	39.6 ± 7.82	38.9 ± 10.64	33.57 ± 7.06	37 ± 6.59	0.54
RBMT delayed	42.47 ± 13.3	37.42 ± 11.63	48.2 ± 7.25	39.33 ± 10.25	34.8 ± 8.37	41.4 ± 10.01	0.33
Attention/Working Memory							
VTS-RT1	47.36 ± 6.53	46.14 ± 8.07	49.41 ± 2.51	46.47 ± 6.32	48.57 ± 7.91	53.6 ± 8.79	0.15
Stroop word	41.33 ± 8.32	42.28 ± 5.49	38.2 ± 7.85	39.19 ± 8.89	39.71 ± 5.61	37.8 ± 8.75	0.81
Stroop colour	44.04 ± 9.88	43.14 ± 7.28	42.4 ± 11.84	38.95 ± 7.76	42.42 ± 11.83	39 ± 13.54	0.75
Stroop interference	44.8 ± 9.42	45.42 ± 6.39	38.4 ± 4.87	39.8 ± 9.52	41 ± 8.2	42.6 ± 7.76	0.80
TMT A	37.09 ± 10.47	41.85 ± 7.31	37.8 ± 12.75	37.95 ± 8.82	41.4 ± 10.7	38.2 ± 20.31	0.32
TMT B *	37.8 ± 12.04	45.14 ± 10.73	45.2 ± 7.66	37.66 ± 14.18	38.57 ± 12.98	40.8 ± 16.78	0.63
TMT B/TMT A	1.01 ± 0.25	1.10 ± 0.31	1.27 ± 0.32	1 ± 0.39	0.96 ± 0.4	1.2 ± 0.34	0.60
DS-WAIS III	11 ± 3.54	10.57 ± 4.54	10.4 ± 4.61	9.9 ± 3.54	10.85 ± 4.18	10.2 ± 4.65	0.94
Semantic and Phonetic Fluency (Executive Retrieval)							
Semantic fluency	50.88 ± 8.39	52.35 ± 8.21	47.8 ± 6.02	45.33 ± 9.51	46.68 ± 11.72	48.7 ± 13.96	0.71
Phonetic fluency	48.61 ± 10.16	51 ± 12.97	42.6 ± 8.29	43.8 ± 12.82	45 ± 15.3	45 ± 5.24	0.95

Abbreviations: *AVLT* = Dutch version of Rey’s Auditory Verbal Learning Test; *RBMT* = Rivermead Behavioural Memory Test; *VTS-RT1* = Single Choice Reaction Time Measurement of Vienna Test System; *TMT A* = Trail-Making Test part A; *TMT B* = Trail-Making Test part B; *DS-WAISIII* = subtest Digit Span of the Wechsler Adult Intelligence Scale III. * TMT-B also informs cognitive flexibility.

## Data Availability

Data are available upon request.
